# Public preferences for the allocation of donor organs for transplantation: Focus group discussions

**DOI:** 10.1111/hex.13047

**Published:** 2020-03-18

**Authors:** Carina Oedingen, Tim Bartling, Marie‐Luise Dierks, Axel C. Mühlbacher, Harald Schrem, Christian Krauth

**Affiliations:** ^1^ Institute for Epidemiology Social Medicine and Health Systems Research Hannover Medical School Hannover Germany; ^2^ Center for Health Economics Research Hannover (CHERH) Hannover Germany; ^3^ Institute of Health Economics and Health Care Management Hochschule Neubrandenburg Neubrandenburg Germany; ^4^ Duke Department of Population Health Sciences and Duke Global Health Institute Duke University Durham NC USA; ^5^ Department of General, Visceral and Transplant Surgery Medical University Graz Graz Austria; ^6^ Transplant Center Graz Medical University Graz Graz Austria

**Keywords:** attitudes, distributive justice, focus group discussion, organ allocation, organ transplantation, preferences, public perspective

## Abstract

**Background:**

Deceased donor organs are scarce resources because of a large supply‐and‐demand mismatch. This scarcity leads to an ethical dilemma, forcing priority‐setting of how these organs should be allocated and whom to leave behind.

**Objective:**

To explore public preferences for the allocation of donor organs in regard to ethical aspects of distributive justice.

**Methods:**

Focus groups were facilitated between November and December 2018 at Hannover Medical School. Participants were recruited locally. Transcripts were assessed with content analysis using the deductive framework method. All identified and discussed criteria were grouped according to the principles of distributive justice and reported following the COREQ statement.

**Results:**

Six focus groups with 31 participants were conducted. Overall, no group made a final decision of how to allocate donor organ; however, we observed that not only a single criterion/principle but rather a combination of criteria/principles is relevant. Therefore, the public wants to allocate organs to save as many lives as possible by both maximizing success for and also giving priority to urgent patients considering the best compatibility. Age, waiting time, reciprocity and healthy lifestyles should be used as additional criteria, while sex, financial status and family responsibility should not, based on aspects of equality.

**Conclusions:**

All participants recognized the dilemma that prioritizing one patient might cause another one to die. They discussed mainly the unclear trade‐offs between effectiveness/benefit and medical urgency and did not establish an agreement about their importance. The results suggest a need of preference studies to elucidate public preferences in organ allocation.

## 1 INTRODUCTION

Transplantations are widely accepted as the treatment of choice for patients with end‐stage solid organ disease and are known to improve the chances of long‐term survival as well as the quality of life.^1^, ^2^ Unfortunately, the demand for deceased donor organs substantially exceeds supply all over the world. A major challenge is to make decisions regarding how these scarce resources should be allocated and who should be considered to receive an available organ.^3^ The resulting decision making and priority‐setting is an expression of the ethical dilemma caused by the organ scarcity as well as the life‐and‐death situation, forcing value judgements between different wait‐listed patients as potential recipients.^3^, ^4^

In most countries, factors such as time on the waiting list, medical urgency, probability of transplantation success and age under 18 years are used as allocation criteria.^5^ The objectives of medical urgency and probability of success can be conflicting, however, since success rates of transplantations typically decrease when urgency increases.^6^ Overestimation of the value of post‐transplant success may lead to an unacceptable denial of transplantation for patients with the highest urgency, and it may put patients who could also live without transplantation with an acceptable prognosis at an unnecessary perioperative risk.^7^

There is a continuous debate about the appropriate choice and relative weighting of various allocation criteria and their impact on fairness.^8^, ^9^, ^10^ Therefore, allocating donor organs is a societal task in which not only medical professionals and transplant patients are important key stakeholders, but also the general public. A public consensus on priority‐setting in organ allocation is of high relevance because deceased donor organs are a public resource as the supply of organs is dependent on public willingness to donate. It should be noted that individuals donate their organs for transplantation anonymously and altruistically without any possible return, whereby public acceptance of allocation rules and criteria is an important prerequisite for consent to post‐mortem organ donation during a person's lifetime. Furthermore, public preferences can be used to inform policy in order to warrant socially responsible allocation systems.^11^

### 1.1 Aims

The aims of this study are (a) to explore public preferences for the allocation of donor organs, (b) to gain better insights into the views and explanations whether and how the public wishes to differentiate between different wait‐listed patients as potential recipients and (c) to understand their perspectives on how this public resource should be allocated. This study is part of a wider project on preferences in organ allocation.^12^ The preferences used were categorized into a theoretical framework of distributive justice principles which were systematically linked in a systematic review preceding this study, which in return is used to verify the results of the review.^11^

### 1.2 Theoretical framework: principles of distributive justice

Donor organs are allocated worldwide by institutions such as Eurotransplant or the National Health Service Blood and Transplant to recipients without expected return, such as costs or prices. In the consequence, organ trading is internationally banned as criminal activity. Therefore, the allocation procedure by the institutions should match ethical aspects of distributive justice in order to guarantee the best ethically accepted allocation system. In this context, distributive justice is best thought of as *‘providing moral guidance for the political processes and structures that affect the distribution of benefits and burdens in societies’*.^13^ In organ allocation priority‐setting, we identified different principles of distributive justice: egalitarianism (treating people equally), utilitarianism (maximizing total benefits), favouring the worst‐off (severity of illness/social disadvantages), own fault (demoting and punishing irresponsibility) and value for society (promoting and rewarding social usefulness). Additionally, the medical background and sociodemographic status may impact both effectiveness/benefit and medical urgency. Therefore, these two groups present medical and social risk factors influencing allocation (see Table 1).^11^

**Table 1:** Theoretical framework: principles of distributive justice (derived from earlier publication^11^)

Medical and social risk factors for effectiveness/benefit and increased urgency		Principles of distributive justice	
Medical background	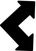	Effectiveness/Benefit (utilitarianism)	Theory‐guided groups: Divergence from principle of equality (egalitarianism)
Sociodemographic status		Medical urgency (favouring the worst‐off)	
		Own fault	
		Value for society	

## 2 METHODS

### 2.1 Study design

This study chose a qualitative design, using focus group discussions, which unlike quantitative surveys offers a deep insight into participants’ views. People's preferences can change after a period of discussion and consideration.^14^, ^15^, ^16^ Therefore, it is important to provide suitable time to discuss the issues and also to reconsider. Furthermore, this design allows analysis of participants’ thoughts and interpretations about different allocation criteria as well as how they interrelate them.

Based on a prior systematic review^11^ and discussion among the researchers, we developed a discussion schedule which could be used flexibly with three phases: (a) introductory phase about thoughts and attitudes to organ transplantation and allocation, (b) criteria which should be considered and how recipients should be prioritized and (c) a ranking exercises of the criteria identified from phase two (see Table 2 for example and Appendix S1 for the complete version). The ranking exercise used a modified nominal group technique.^17^, ^18^, ^19^ This approach was only used in order to receive more information about the relative importance of the discussed criteria for the participants, with the possibility to elucidate a possible group consensus. We pre‐tested the discussion schedule in November 2018.

**Table 2:** Discussion schedule

	Topic	Example questions
(a)	Preliminary questions about general thoughts and attitudes to organ transplantation and allocation	To what extent is your knowledge about organ allocation in Germany? What comes first to your mind about the issue of organ allocation respective distribution?
(b)	Group discussion on what criteria should be considered in organ allocation and how recipients should be prioritized to receive organs	If you were to decide, what criteria should be used to allocate deceased donor organs? Do seem all criteria equally important to you? How do you assess the criteria as medical urgency and effectiveness/benefit for the allocation of donor organs? What would be of importance to you when it comes to allocating your own organs after death?
(c)	Ranking exercise of the criteria identified from the group discussion	The participants were asked to individually start ranking the criteria by using their five stickers. After each participant allocates their stickers, we sorted the criteria by the respective number of stickers and asked the participants if they were satisfied with the group ranking and gave the possibility to discuss again some ambivalent criteria.

Maximum variation sampling was applied. Therefore, participants were eligible if they spoke and understood German, were at least 18 years old and able to give informed consent. We excluded transplant patients and medical transplant professionals because their perspectives likely differ significantly from those of the public due to fundamentally different interests. The participants were sorted in different groups according to their level of information and experience with organ transplantation and allocation: one group with participants who do not have any information/experience, one who do, and a third, mixed group. Information and experience were defined as knowing someone who needs organ transplantation or who is already transplanted (relatives, friends or colleagues) as well as being occupationally involved in health care. Overall, we conducted six focus groups, two for each level and two mixed. The participants were purposively sampled to ensure a balance of gender, age and cultural backgrounds, when possible. Participants were offered reimbursement for their travel expenses. The study was approved by the Hannover Medical School Human Ethics Committee (Vote number: 7921_BO_K_2018).

### 2.2 Recruitment and participants

We recruited participants both through an event series ‘patient university’ at Hannover Medical School, Germany, and through local online advertising. ‘Patient university’ is an independent health education institution to increase health literacy in the public, which organizes free events to facilitate a health‐and‐illness–related discussion between the public and health professionals.^20^ We had an information booth where we directly invited people to participate in our study in October and November 2018. The online advertisement was carried out via websites of the Hannover Medical School and the city of Hannover. Most of the participants (N = 25) applied through the patient university. During the recruitment, we used a short questionnaire that asked about the level of information/experience for our group selection.

### 2.3 Data collection

The focus groups were facilitated between November and December 2018 at Hannover Medical School and lasted for approximately two hours. They were facilitated by CO. Due to quality criteria for group discussions,^21^ the facilitator posed queries, provoked discussion, moderated a respectful dialogue between the participants and assured that all participants had the opportunity to get involved in the discussion. TB visualized the relevant discussed criteria on a flip chart as well as recorded field notes on group dynamics and interactions, participant characteristics and the context surrounding the discussion. Every discussion ended with a short questionnaire. All participants received verbal and written information about the study and had the possibility to withdraw at any time. Participants gave consent to audio taping and the use of anonymized quotes. All sessions were recorded and transcribed verbatim for analysis.

### 2.4 Data analysis

The transcripts were assessed with content analysis using the deductive framework method^22^ to identify major themes associated with preferences for the allocation of deceased donor organs. CO and TB read and reread all transcripts. A coding scheme was developed by each researcher individually based on a deductive category system according to the principles of distributive justice.^11^Sections with relevant content were coded on a sentence‐by‐sentence basis and applied to deductive categories. Once a category had been identified and coded, further examples were coded and added to the category system only if they extended its meaning. While coding all transcripts, the coding scheme was regularly discussed and adjusted based on new findings. The transcripts were analysed with MAXQDA Plus 2018 (VERBI Software GmbH V.18.2.0), and the findings are reported following the Consolidated Criteria for Reporting Qualitative Research (COREQ) statement.^21^

## 3 RESULTS

### 3.1 Participants characteristics

All focus groups included a total of 31 participants involving four to seven participants in each group. Most of them were female (61.3%); the average age was 55.9 (21‐83) years, and the majority had no information/experience previously about organ transplantation and allocation (67.8%). Most of the participants reporting experience also knew someone who had already received a donor organ. About half (48.4%) of the participants reported that they have a signed organ donor card, whereas most of them had information/experience with the topic (see Table 3).

**Table 3:** Characteristics of participants

	Total (%)	Group 1 inexperienced	Group 2 mixed	Group 3 with experience	Group 4 mixed	Group 5 inexperienced	Group 6 with experience
Total	31 (100)	6	5	4	5	7	4
Sex							
Male	12 (38.7)	2	1	2	2	3	2
Female	19 (61.3)	4	4	2	3	4	2
Age group^a^							
<34 y	4 (12.9)	1	0	1	2	0	0
35‐54 y	7 (22.6)	0	2	0	1	2	2
>55 y	19 (61.3)	4	3	3	2	5	2
Organ donor card							
Yes	15 (48.4)	1	4	3	3	1	3
No	16 (51.6)	5	1	1	2	6	1
Organ transplantation experience							
Private experience	5 (16.1)	0	1	2	0	0	2
Professional experience	5 (16.1)	0	0	2	1	0	2
No experience	21 (67.8)	6	4	0	4	7	0
Knowing transplant patients in social surrounding							
Yes, waiting for donor organ	1 (3.2)	0	0	0	0	0	1
Yes, received donor organ	6 (19.4)	0	1	2	1	0	2
No	24 (77.4)	6	4	2	4	7	1

^a^ Due to the fact that a participant probably skipped this field, a value is missing.

### 3.2 Thematic synthesis

Depending on the level of information/experience, most of the participants had only little knowledge of current organ allocation policy, but all of them recognized that donor organs are a scarce resource forcing value judgements between different wait‐listed patients. Therefore, they discussed the need for consideration of various criteria in allocation; however, they found it difficult to decide between equally important criteria resulting in inconsistent preferences. When comparing hypothetical patients (either defined by the facilitators or the participants themselves), they often struggled to prioritize one over the other. Thus, in all focus groups the participants could not agree about which criteria to use to allocate donor organs. They wanted donor organs to be treated responsibly and transplanted into the most urgent recipients with the potential for the best possible outcomes. According to our framework, we categorized the preferences into egalitarianism, effectiveness/benefit, medical urgency, own fault, value for society, medical background and sociodemographic status. In the following, the preferences were summarized and underpinned with direct quotations.

#### 3.2.1 Egalitarianism

All participants thought that donor organs should be allocated fairly and equitably. Because organs are ‘indivisible’, egalitarianism means the provision of equal chances of transplantation rather than equal amounts of it.^11^

Therefore, everybody should have the equal right and should be guaranteed equal “chances” of access to a donor organ. (ID14, FGIII, with experience)

Participants with experience saw egalitarianism on an abstract level which has to be achieved long‐term, and they understood that not every patient who needs an organ will be transplanted. In this case, other criteria are relevant for the allocation diverging from the principle of egalitarianism. Particularly, participants without experience wanted to avoid this dilemma and instead suggested rolling a dice, coin flipping or lottery. Egalitarianism includes also the time on the waiting list, because when other criteria such as medical urgency or effectiveness/benefit were equal, this criterion was considered as quite fair. Overall, the discussion was always about the fairness of allocation.

[…] What am I supposed to choose? I wouldn't know, it's either flipping a coin or taking time on the waiting list into consideration, what's fair now? (ID3, FGI, without experience)

#### 3.2.2 Effectiveness/Benefit

The benefit from transplantation was an important criterion for all focus groups and was discussed in terms of increasing life as well as benefitting quality of life to reduce the risk of lost opportunities. Therefore, before an organ was offered to a recipient, participants wanted to ensure that there is a reasonable potential to survive surgery and to live as long as possible without a rejection of the donor organ. Failed transplantation, especially at the stage of the surgery, was not only seen as a loss for the recipient, but also as a lost opportunity for another wait‐listed patient.

[…] We certainly have to deal responsibly with this scarce good and we must try to give life or many years of life. (ID6, FGI, without experience)

I would also say that success is relevant, otherwise it makes no sense. Yes, of course, that would be a wasted organ that could have helped someone else. (ID21/22, FGV, without experience)

They considered the short‐term operative survival as well as the overall life expectancy, but did not discuss the variation in benefits and made no operationalization at which point benefits were maximized. They discussed more often, however, that the survival could not be as accurately predicted and hence remained still vague.

Only in the focus groups with experience, a discussion of what life would be like with a new organ emerged. While participants without experience discussed solely the maximization of life years, these groups also discussed the benefit in post‐transplant quality of life.

[…] The consolidation of the status quo and therefore keeping someone in bad health should not be the goal [of transplantation]. (ID15, FGIII, with experience)

#### 3.2.3 Medical urgency

Besides the maximization of effectiveness/benefit, all focus groups wanted to consider patients who need an organ most urgently to prevent fatal consequences or further organ damage. This was mainly discussed in terms of pre‐transplant life expectancy and quality of life. Only the focus groups with experience discussed the quality of life while waiting for a donor organ and concluded that it should be incorporated as an add‐on in the evaluation. Overall, participants wanted to save as many patients as possible and not lose anyone while they were waiting for an organ:

The patient, who only has six months left to live, is preferred over the patient who can wait one or two more years. (ID17, FGIV, mixed)

Most of the participants could define urgency easier than effectiveness/benefit because many believed that those who were in immediate danger of death or who were the sickest should be of highest priority.

Urgency being factual data rather than estimations should give it a higher priority. (ID16, FGIV, mixed)

In all focus groups, the participants wanted to consider the time on the waiting list only as an additional criterion. According to the results of the systematic review, we clustered waiting time in medical urgency because the public associated it with an increase of urgency in the included studies.^11^ Patients typically became more impatient with increasing waiting time, and those who were on the waiting list for a longer time often had a high urgency for a new transplant. This view was also discussed in the focus groups with experience, and therefore, the waiting time was seen as an urgency aspect, despite this paradox:

[…] Patients listed already have a certain progression of illness. However, it might be that they have less priority than someone else with a higher severity. This leads to them not getting organ offers yet, but at a later time, where then again their chances of success are worse. (ID15, FGIII, with experience)

As opposed to this, participants without experience felt that priority should be given to those who had been on the waiting list the longest, as this was rated under fairness aspects. Potential recipients waiting for a long time because more urgent or more suitable patients were preferred for transplantation triggered a feeling of injustice:

I personally perceive it as unjust that someone waiting for an organ for a long time always gets surpassed on the waiting list by higher urgency cases or cases with better compatibility, until that person is not transplantable anymore. (ID6, FGI, without experience)

#### 3.2.4 Own fault

The discussion of health‐related behaviours and individual role in causing the failure provoked dissent and caused the most moral discomfort between all focus groups. Some participants were very in favour of taking some lifestyle factors such as alcohol abuse, smoking, illicit drug use or non‐compliance into consideration. They did not necessarily think that potential recipients with these lifestyle habits were deserving of an organ:

[…] Some people are sick by birth and need a new organ, while others need an organ due to negligence. Sometimes, people take no responsibility for the own health status. […] (ID27, FGV, without experience)

[…] While you shouldn't exclude alcoholics, smokers and drug abusers per se, it is perfectly justifiable to disregard these people due to their worse prognosis after transplantation, which makes an exclusion of this group ethically understandable. (ID6, FGI, without experience)

Other participants were very against this. They both voiced concerns about how to differentiate between when someone can be held accountable for their actions, and when not, and additionally felt that patients should be given a second chance and may be given the opportunity for everyone to change their lifestyle after transplantation.

When being around many smokers, you can get lung cancer due to second‐hand smoke. Therefore, it is very difficult to take responsibility for someone's own life into account. […] (ID20, FGIV, mixed)

[…] Facing nearly certain death, some patients start to rethink because the rest of their life is too valuable to destroy it any further. (ID13, FGIII, with experience)

#### 3.2.5 Value for society

Some focus groups discussed the extent of family responsibility in caring for children or other dependents and the community value such as occupational role or volunteering. Participants found it difficult, however, to judge these criteria, making them unable to use. Instead, all focus groups raised the idea of reciprocity: the participants wanted to give priority for recipients who were also prepared to be a donor themselves before their organ failure. They discussed that reciprocity would lead to more solidarity, appreciation and fairness in the organ allocation process. Moreover, this procedure can also contribute to tackle the lack of donor organs.

…It would be the most fair if only people get organs who also want to donate themselves. This would be consequent. […] Someone who does not want to donate an organ, does not get one because of the missing solidarity. Bad luck. (ID13, FGIII, with experience)

[…] prefer people who are willing to donate themselves because it would honour their choice for donating their own organs and consequently help that more people are filling out an organ donor card. (ID17, FGIV, mixed)

During the discussion, some participants saw problems with some groups not being able to give consent to organ donation before their own need for an organ, such as children or persons with dementia.

#### 3.2.6 Medical background

One of the first addressed criteria was the medical compatibility between donor and recipient, such as blood type compatibility, tissue matching, height and weight, as this would reduce the chance that donor organ function might fail after transplantation. Rejection was viewed as a missed opportunity, and some participants argued that a perfect match also increases the chances of transplantation success. Overall, this criterion was seen as a necessary condition and therefore non‐negotiable:

[…] first, the donor organ has to match, this is a yes or no decision. If it does not match, then I do not need to carry on. (ID17, FGIV, mixed)

To some extent, the donor and recipient should also be matched on age to increase the durability of the organ and to reduce the number of transplants needed over life. As a further lost opportunity, the participants discussed that the potential recipient should be in good enough health condition to survive the transplantation and not to die of another disease. This was also seen as a possibility to increase the effectiveness/benefit.

…not only the sick organ, but rather the whole health of the recipient should be of relevance. (ID11, FGII, mixed)

#### 3.2.7 Sociodemographic status

Participants did not want to allocate donor organs inherently after the recipient's age. But they thought that a younger age is associated with a longer life expectancy and thus leads to greater transplant benefit; therefore, age should be considered as a surrogate parameter for effectiveness/benefit. They discussed that younger recipients have a better general health status before transplantation, can accept a donor organ easier and have a better life expectancy and quality of life after transplantation. Some of the participants argued that under equality aspects children and adolescents should be prioritized because they have had a shorter life. Overall, many had difficulties defining a value for the age range that should have priority.

The transplantation success rate is certainly better for a younger recipient than an older recipient. […] (ID27, FGV, without experience)

[…] If we have the choice between a younger and older recipient then it makes maybe more sense, under ethical or social aspects, to give priority for the younger. (ID28, FGVI, with experience)

[…] I think we all agree that adolescents and children should be prioritized because they still have their whole life ahead of them. (ID14, FGIII, with experience)

The only consensus to be reached is that donor organs should not be allocated by sex or financial status. In these cases, all potential recipients should have the same chance.

### 3.3 Identified trade‐offs for the allocation decision

Besides the discussions of relevant criteria, the focus groups also identified some trade‐offs inherent for the decision of organ allocation. The participants discussed mainly the trade‐off between the principles of effectiveness/benefit and medical urgency, even if not every participant was aware of this trade‐off. They identified two potentially conflicting goals in this trade‐off: the decision to maximize the outcome from a donated organ and the decision to save all patients in need of a donor organ. They defined the outcome primarily as post‐transplant life expectancy and the medical need as pre‐transplant life expectancy. It was discussed how to balance the contrary principles of success rates and urgency, since an increase in one of these factors always implies a decrease in the other:

Often, effectiveness and urgency do not go hand in hand, therefore exclude each other. […] (ID31, FGVI, with experience)

But I think the two criteria urgency and chance of success are rather difficult. If I have a patient who needs the organ urgently, but the chances of success are totally low. How should I decide? […] (ID16, FGIV, mixed)

Particularly, participants with experience defined the principle of effectiveness/benefit after transplantation not only in terms of life expectancy, but also in terms of quality of life, which needed to be considered and balanced against the expected length of survival following the transplantation:

I think we all agree that we have so few organs that it makes no sense to transplant a donor organ and the recipient has no improvements in quality of life, while another patient dies waiting for it. (ID14, FGIII, with experience)

In these discussions, they subsumed criteria as age, individual role, waiting time and compliance under aspects of transplantation success or medical urgency, respectively, showing that many criteria are overlapping and therefore not clearly definable:

[…] Of course, the patient has less chance of success if he continues to smoke. If he gets a new lung and keeps smoking, he has less chance of success. (ID24, FGV, without experience)

If we consider the life expectancy between a 40 year old recipient and 70 year old recipient, the benefit of the organ after transplantation is quite different. The younger recipient is expected to benefit more from the organ. (ID8, FGII, mixed)

Overall, all trade‐offs showed that the participants have to base their value judgements on moral beliefs and fairness. Therefore, in all trade‐offs moral values have to be weighed against the identified and relevant criteria, leading to the result that fairness aspects are included in all judgements.

## 4 DISCUSSION

These are the first focus group discussions of this kind. Overall, no group could establish how to allocate organs and decide which criteria should have higher priority; however, the discussions had identified a considerable number of criteria and trade‐offs that influence these preferences. All focus groups concluded that no single criterion should be used as the over‐riding respective principle, but rather a combination of different criteria respective principles are relevant for the allocation decision. They assessed that donor organs are scarce resources and should be handled with care, forcing value judgements between different wait‐listed patients. Thus, it seemed obvious that when a patient is prioritized, someone else will be disadvantaged and consequently may die without transplantation in time while their disease has progressed further. Participants expressed that medical compatibility between donor and recipient is a mandatory criterion which has to be met before further criteria of effectiveness/benefit and medical urgency should be taken into consideration. They wanted to save as many lives as possible in order to lower the risk for lost opportunities especially for those on the waiting list by maximizing transplant success, but also wanted to give high priority to urgent patients facing imminent death. Criteria such as waiting time, lifestyle factors, reciprocity, medical background and sociodemographic status should be used as additional or surrogate criteria, but not as criteria singlehandedly. Sex, financial status or family responsibility should be covered by the principle of equality.

The focus group discussions identified mainly the trade‐off between effectiveness/benefit in terms of expected survival and medical urgency in terms of imminent death while this trade‐off could not be quantified. We tried to discuss the measurements of these conflicting criteria and gave different examples between hypothetical recipients with the aim to compare and figure out at which point the public gives higher priority for one criterion or the other. Unfortunately, in all focus groups the public stated that either they need more information about the potential recipients to reach an agreement, participants switched to other criteria which suddenly became relevant although they considered them irrelevant at the beginning of the discussion, or they stated that they did not want to make a final decision on such a life‐and‐death trade‐off. These various arguments show the public's ethical dilemma: taking one side means disadvantaging the other side and therefore creating a group of patients that are considered of less priority than others. Participants’ refusal to giving a final answer is likely the result of the understandable human propensity to avoid hard choices whenever possible. That is also the reason why no agreement could be identified and sometimes inconsistent preferences occurred. Furthermore, in our previous systematic review, we were able to identify numerous trade‐off statements that were hardly mentioned in the studies included in the review.^11^ The same trade‐offs were observable in the current qualitative work, additionally confirming our assumptions.

Separating the groups with experience from the ones without experience, quantitative lifetime before and after transplantation was considered as well as taking quality of life into account. This can be most likely ascribed to the personal knowledge of and experience with transplant patients. We have recently identified similar preferences for the group of professionals: physicians give less priority to criteria if they could lead to a less successful transplantation outcome.^23^ Therefore, the extent of personal knowledge and experience has a relevant influence on the consideration of quantitative as well as qualitative success criteria. We were surprised that for all participants the most requisite criterion was the medical compatibility between donor and recipient regardless of the level of experience. They believed in the impact of compatibility and the small probability to find the perfect match, whereby the allocation can be made easier. We argue that in general the public is less knowledgeable about medical issues but they nevertheless assign them a high relevance with respect to organ allocation. This is even more astonishing considering that the allocation is morally and ethically complex with considerable uncertainty and most of the discussed criteria are not inherently medical (eg waiting time, reciprocity or age). These observations lend some weight to a psychological explanation: the participants strive to avoid an ethical dilemma by delegating it to medical experts, who are expected to produce medical criteria of biological compatibility as the *conditio sine qua non* that shields the public from having to take a position in this dilemma, and which forces a decision leading to individual casualties due to the scarcity of organs. This propensity is indeed surprising, and no effective solution was identified. The obvious approach to alleviate this dilemma would be to optimize organ donation with the goal to improve the currently very low donation rates in Germany.^24^, ^25^

Information about the patient's lifestyle and self‐inflicted behaviour such as drinking alcohol or smoking was relevant to balance the allocation decision but led to controversial discussions. Participants felt that these criteria are discriminatory because alcohol abuse or smoking is an addiction and therefore an illness; however, they discussed that it may not be ethical to prefer a patient who is responsible for their organ failure when this implies that another patient who is not responsible would not receive an organ. From a public perspective, organ failure caused by alcohol consumption or cigarette consumption is considered as attributable to lower post‐transplantation survival rates. Therefore, some participants wanted to avoid this dilemma and used lifestyle criteria as a surrogate parameter for effectiveness/benefit although today's survival rates between patients with and without such behaviour are very similar.^26^ Nevertheless, most of the current allocation policies require a drinking or smoking cessation of at least six months before liver or lung transplantation, respectively. This is done in order to allow the patient's organ a chance to recover and to reduce the risk of consumption relapse.^26^, ^27^, ^28^, ^29^, ^30^ Overall, all criteria which are normally culturally accepted under equality aspects were seen as surrogate parameters for effectiveness/benefit to avoid the dilemma of discrimination against some subgroups which have an unhealthy behaviour or an older age.

All focus groups discussed the organ allocation system under fairness aspects. It was discussed that the system has to be just, resulting in an equitable and appropriate process of distribution for the whole society. This implies not only the patients who need organs or the transplant professionals who work in this field but rather the public who donate their organs anonymously and altruistically. Fairness aspects were consistently inherent during the discussions resulting among others in the possibility of reciprocity by prioritizing recipients who have been registered donors prior to their organ failure, as is legitimate in Israel.^31^ The participants justified such a rule on the basis that it is fair and will encourage donor registration because ‘free‐riders’ are willing to take an organ while they would likely reject to donate an organ themselves. Furthermore, in the beginning all focus groups related fairness with the German ‘transplantation scandals’ in the years 2011 and 2012 during which data were manipulated in hospitals to move the patients up the waiting list for livers. As a consequence, the trust of the public about fairness in organ allocation and donation has been destroyed.^32^, ^33^, ^34^, ^35^

### 4.1 Comparison with previous findings

These results are generally consistent with previous qualitative results,^11^ in which most of the criteria were discussed resulting in preferences to save as many lives as possible by maximizing the transplant success while also giving high priority to urgent patients. It was interesting to recognize, however, that our study was the only one which investigated these preferences without specifying the organ, while the other studies focused on kidney^36^, ^37^ or liver,^38^ respectively. We state that for the public the specific organ is not as relevant in the discussion. For the first time, the participants in our focus groups discussed also the medical compatibility between donor and recipient and believed that this is the requisite criterion that sets the condition before any other allocation considerations come into play. Furthermore, the criteria about ‘own fault’ were discussed very ambivalently and some of the participants wanted to consider these criteria as surrogate parameters for effectiveness/benefit while in the other studies these social judgements were not made.^36^, ^37^, ^38^ Interestingly, family responsibility played a role in the two qualitative studies from the UK,^36^, ^38^ while an Australian study^37^ and ours clearly showed that this criterion should be used under equality aspects.

### 4.2 Strengths and limitations

Qualitative methods are suitable for understanding people's attitudes, opinions, values and perspectives and therefore enable capturing the importance of the data rather than the frequency of responses. We used a coding scheme based on a deductive category system according to the principles of distributive justice. These strengths, however, also lead to some limitations. First, we cannot be sure that the same results would have been obtained from face‐to‐face interviews. There may be a danger that some respondents may have given responses that they thought their group members wanted to hear.^39^ Second, although we tried to apply a maximum variation sampling and a clustering in different groups, our sample was on average 56 years old so that younger people were underrepresented. Third, we can suppose that people especially interested in the topic of organ allocation and transplantation participated so that we have a possible information bias in the findings. This could be due to the fact that most of the people wanted to discuss about their own choice on organ donation. Moreover, the media presented a new bill for an opt‐out system in organ donation in Germany during the data collection process. Fourth, during the discussions, additional themes occurred such as the constitution of an independent council or logistical challenges with the transplantation, whereas reporting these in detail would go beyond the scope of this paper.

## 5 CONCLUSIONS AND FURTHER STEPS

In all focus group discussions, the participants knew the dilemma resulting in value judgements between potential organ recipients and the fact that prioritizing one patient likely puts another patient into an increased risk. They did not, however, come to agreement owing to the understandable human propensity to avoid hard choices that would generate disadvantages for certain patients. If the reported results and identified trade‐offs were to be quantified in a discrete choice experiment, which is able to elucidate and weight the unclear trade‐offs, predominantly those between effectiveness/benefit and medical urgency, recommendations towards a legal framework with higher public acceptance might be obtainable.

## CONFLICT OF INTERESTS

Carina Oedingen and Tim Bartling were funded by the German Federal Ministry of Education and Research. The funding source had no role in the study and no influence on the data collection and analyses, interpretation of the results or writing of publications. Marie‐Luise Dierks, Axel C. Mühlbacher, Harald Schrem and Christian Krauth have no conflicts of interest that are directly relevant to the content of this article.

## AUTHOR CONTRIBUTIONS

CO contributed to the development of the study design, carried out the focus group discussions and analysis, and drafted and improved the manuscript. TB carried out the focus group discussions and analysis, and reviewed and commented on the preliminary drafts and final version of the manuscript. M‐LD, ACM and HS reviewed and commented on the preliminary and final manuscript drafts. CK contributed to the development of the study design, reviewed and commented on the analysis, and reviewed and commented on the preliminary manuscript drafts and the final version. All authors agreed to be accountable for all aspects of this work, ensuring the integrity and accuracy of this paper. All authors revised the manuscript and approved it for publication.

## ETHICAL APPROVAL

The study has received ethics approval from the Hannover Medical School Human Ethics Committee (Vote number: 7921_BO_K_2018). We obtained informed written consent from all participants and followed the ethical guidelines of the World Medical Association Declaration of Helsinki.

## Supporting information

Appendix S1

## Data Availability

The data that support the findings of this study are available on request from the corresponding author. The data are not publicly available due to privacy or ethical restrictions.
