# Supplementary material for: Public preferences for the allocation of donor organs for transplantation: Focus group discussions
Source: Health Expect. 2020 Mar 18;23(3):670–80. doi: 10.1111/hex.13047 (PMC7321724; doi:10.1111/hex.13047)
Supplement: Supplementary file 1 — Appendix S1 [file HEX-23-670-s001.docx]

**Discussion schedule for focus group discussions of the general public**

| **Organisation** | | Time (in min) |
| --- | --- | --- |
| - Prepare the room (refreshments & equipment (min. two flip charts, stickers, cardboard, pens, forms, recording equipment)) - Hand out name tags - Five stickers & forms for each participant - Display main question on the flip charts - „Topic drawer“ | | n/a |
| **Welcoming** | | n/a |
| - Welcoming participants before the discussion - Offer refreshments | |  |
| **Introduction** | | 10 |
| **Introduction** | - Thank the participants for their participation - Short introduction of the facilitators   *My name is … and my colleague here is …. We are both working on a project of the Center for Health Economics Research Hannover, short CHERH, regarding preferences of organ allocation. The CHERH is funded by the Federal Ministry of Education and Research which incorporates health economics researchers by both Hannover Medical University and Leibniz University Hannover. The main goal of CHERH is to improve public health in Germany.* |  |
| **Aims** | *Our goal today is to understand how representatives of the general public want to allocate deceased donor organs. Therefore, we will conduct a focus group discussion, supported by guidelines, in order to exchange ideas and opinions.* |  |
| **Informed consent** | *First, some organisational things: We will be recording the discussion via audio. We guarantee you a discrete handling of the recordings and your personal data. In order to being able to record this discussion, we need your informed consent. Please fill out the statement of agreement in front of you right now.*  *Furthermore, we would ask all participants kindly to handle the things discussed here today discretely. At the end of the discussion I would kindly ask you to fill out and return the questionnaire in front of you.* |  |
| **Conversational rules** | *I would like to state that it is very likely that different opinions will be voiced here today. It is important to us that there are no right or wrong answers in this discussion. Also I would kindly ask you to turn your cell phones off.* |  |
| **Questions** | |  |
| **1. Introduction of participants**   - Involves every participant from the beginning - Is supposed to create a sense of community | *Spotlight:*  ***Please introduce yourselves. Who are you, where are you from and what is your motivation to take part here today?*** | 10 |
| **2. Introduction of the topic**   - Slowly introduces the participants to the topic of interest | ***What is your knowledge about organ allocation in Germany?*** | 15 |
|  | - *What is the first thing that comes to your mind thinking about the topic of …?* - *Organ donation* - *Organ transplantation* - *Organ allocation* |  |
| **🡪 Participants are encouraged to mention some criteria during the next questions. Prepare to write these criteria down and visualise them on one of the flip charts. In case the participants cluster those criteria in groups, visualise their clustering on the flip chart.** | |  |
| **3. Key questions**   - Main research questions - Will take the most time | ***If you were to decide, what criteria should be used to allocate donor organs?*** *(any solid organ)* | 50 |
|  | - If all criteria appear to be of similar importance, ask for a relative rating between relevant criteria. - If these criteria differ for each organ, investigate why. - How are these criteria supposed to be measured? |  |
|  | ***What is your opinion on the currently used criteria “effectiveness/benefit“ and “medical urgency“?*** |  |
|  | - *Optional: Facilitators explain the concepts of “effectiveness/benefit” and “medical urgency” and their current use in the transplantation context.* |  |
|  | ***What would be of importance to you when it comes to allocating your own organ after death?*** |  |
|  | - *Under what circumstances would you be willing to donate your organ after death? Are these criteria equal to the suggestions from the question before? If not, why?* |  |
| **4. Ranking**   - Identify/create a group consent | If it becomes clear that then discussion does not provide any further, not before mentioned aspects, ask the participants to individually start ranking the criteria by using their stickers. Each participant has a total of five stickers that they are free to allocate to the criteria on the flip chart. It is possible to distribute them amongst one or more criteria to their liking.  After each participant allocates their stickers, the facilitators sort the criteria by the respective number of stickers and present this ranking to the group.  ***Does this result surprise you or do you feel it represents the consent of the group?*** | 20 |
| **Optional:** | ***Who should be able to decide how to allocate the donor organs?*** | n/a |
| **5. Concluding the discussion** | The facilitators give a short conclusion of the discussion.  ***Thinking about the discussion, what turned out to be the most important aspect for you?***  ***Is there anything we should have covered today that we haven’t?***  ***There are going to be more discussions like this in the future. Do you have any recommendation on how to improve?*** | 5 |
| **Wrapping up** | ***We are happy to inform you about the progress of the study. If you want us to contact you any further on this topic, please let us know.*** | 10 |
